# Metal vapor micro-jet controls material redistribution in laser powder bed fusion additive manufacturing

**DOI:** 10.1038/s41598-017-04237-z

**Published:** 2017-06-22

**Authors:** Sonny Ly, Alexander M. Rubenchik, Saad A. Khairallah, Gabe Guss, Manyalibo J. Matthews

**Affiliations:** 1 Materials Science Division, Physical and Life Sciences Directorate, Lawrence Livermore National Laboratory, 7000 East Avenue, Livermore, California 94550 USA; 2 Laser Science and Systems Engineering, NIF and Photon Sciences Directorate, Lawrence Livermore National Laboratory, 7000 East Avenue, Livermore, California 94550 USA; 3 Computational Engineering, Engineering Directorate, Lawrence Livermore National Laboratory, 7000 East Avenue, Livermore, California 94550 USA; 4 Laser Systems Engineering Operations, Engineering Directorate, Lawrence Livermore National Laboratory, 7000 East Avenue, Livermore, California 94550 USA

## Abstract

The results of detailed experiments and finite element modeling of metal micro-droplet motion associated with metal additive manufacturing (AM) processes are presented. Ultra high speed imaging of melt pool dynamics reveals that the dominant mechanism leading to micro-droplet ejection in a laser powder bed fusion AM is not from laser induced recoil pressure as is widely believed and found in laser welding processes, but rather from vapor driven entrainment of micro-particles by an ambient gas flow. The physics of droplet ejection under strong evaporative flow is described using simulations of the laser powder bed interactions to elucidate the experimental results. Hydrodynamic drag analysis is used to augment the single phase flow model and explain the entrainment phenomenon for 316 L stainless steel and Ti-6Al-4V powder layers. The relevance of vapor driven entrainment of metal micro-particles to similar fluid dynamic studies in other fields of science will be discussed.

## Introduction

Hydrodynamic studies of vapor jet streams have been pursued in support of numerous practical applications in science and engineering^1^. Entrainment occurs as the vapor jet interacts with its surroundings, pulling in and accelerating ambient gas toward the jet stream. The ambient gas flow can sweep up particles along its path acting as a primary transport mechanism for a collection of particles. Vapor-driven entrainment of particles has been used to explain important geophysical phenomena^2, 3^, spur new basic energy research^4^, establish novel materials synthesis methods^5^, and for the development of various industrial applications^6^. For example, in material deposition processing, the entrainment of micron-size particles in a gas stream is a basis for new microplasma coating technologies^7, 8^.

Processing of micron scale metal powders is important to several applications in metallurgy (e.g. sintering, cold casting) but also in more recent fields such as magnetorheological polishing, personal body warmers, and metal 3D printing. In the field of metal 3D printing, there is extensive ongoing research in laser powder bed fusion additive manufacturing (PBFAM) process to fabricate high quality, high density metal parts with unique functionality and excellent mechanical properties^9^. The use of high powered lasers to fully melt layers of ~30 μm sized powder also bring about complex hydrodynamics involving multiphase interactions between vapor plume, atmospheric gas and material that are not well understood. During melting of the powder, one can often observe directly very bright particles ejected from the melt pool, having the appearance similar to welding sparks, commonly referred to as spatter^10^. Spatter generation is generally considered to be a detrimental process in PBFAM. Research has shown that droplets ejected can undergo oxidation and change the chemical composition during flight, leading to contamination of the powder bed with different microstructures and particle sizes^11, 12^. A large number of droplets that fall back on the substrate can increase the surface roughness during melting of each layer^13^, enhance process-induced porosity^14^, or increase the layer thickness^15^ resulting in lack of fusion in the built material^16^. Understanding the ejection formation and general fluid-particle interaction during the PBFAM process is important to predict layer quality, morphology, and density of the final part.

The generally accepted explanation for melt pool dynamics and droplet ejection in laser PBFAM is based on understanding of a phenomenon known as recoil pressure^17^. When the temperature of the metal surface approaches and exceeds the vaporization temperature, a metallic vapor jet will be formed. A high recoil pressure (>10^4^ Pa) produces a downward force on the melt pool causing rapid melt pool motion and leading to liquid metal ejected away from the melt pool. Up until now, this recoil pressure model formed the basis for understanding material expulsion from the metallic melt pool in PBFAM processes. However, it was previously shown that the displacement of powder particles through vapor jet entrainment is the principal cause of powder layer denudation (removal) adjacent to a melt track^18^, a surface feature first described by Yadroitsev^19^. Denudation driven void structures are known to degrade metal component properties and performance, requiring careful choice of laser scan strategies for PBFAM builds^20^. While evidence was presented in our previous work to support vapor entrainment-driven denudation, the underlying physics and broader impact on other observables – most notably molten spatter generation – is only now more fully understood.

The central aim of our present study is to elucidate what is realized now to be the driving mechanism for material redistribution in the PBFAM process, thus clarifying the source of several defect-generating phenomena. We will show that the dominant mechanism leading to particle ejection is from vapor-driven entrainment of micro-particles and not vapor recoil pressure as is universally understood. Little attention has been paid on the potential effect of a local entrainment process and no clear description of its effect on material redistribution has been given. Although the physics involved is somewhat specific to PBFAM technology, a better understanding of micro-particle entrainment processes, in particular in the presence of strong pressure and temperature gradients, can help shed light on entrainment in other applications.

The experiments presented here are conducted with 316 L stainless steel (SS316L) and Titanium alloy Ti-6Al-4V (Ti64) powder layers and plates, materials that are relevant to PBFAM. We describe the dominant physical effects associated with micro-droplet spatter generation, the interaction of the powder particles with surrounding gas dominated by an entrainment process, droplet thermal transients and material ejection processes. To help elucidate the experimental results, we model the laser-melt pool interaction with a multiphysics finite element code (ALE3D)^21^. We discuss the combination of the experimental and computational results with simple physical estimates, providing a holistic picture of the hydrodynamic effects related to metal AM and other particle-fluid microsystems.

## Results and Discussion

### Recoil pressure-driven spatter

The underlying mechanisms and effects of melt pool ejection have been investigated extensively in welding processes for a wide range of conditions^22–29^, while studies for PBFAM are more limited^11, 30–32^. Welding – a joining process - uses a relatively slow-moving (or stationary), high energy density laser with a large spot size (~1 mm) to melt a deep channel of a few millimeters on a bare metal plate. PBFAM - a deposition process - uses a fast scanning (~1 m/s), smaller laser beam size (typically 50–200 µm) to melt particles (tens of microns) in a powder bed. Consequently, the melt pool hydrodynamics for both situations can be quite different. To clarify the differences, we recorded ultra-high speed image sequences of the laser-driven melt pool motion for tracks produced on a flat metal substrate and compare them to those recorded for a substrate covered by a layer of metal powder.

**Figure 1:**
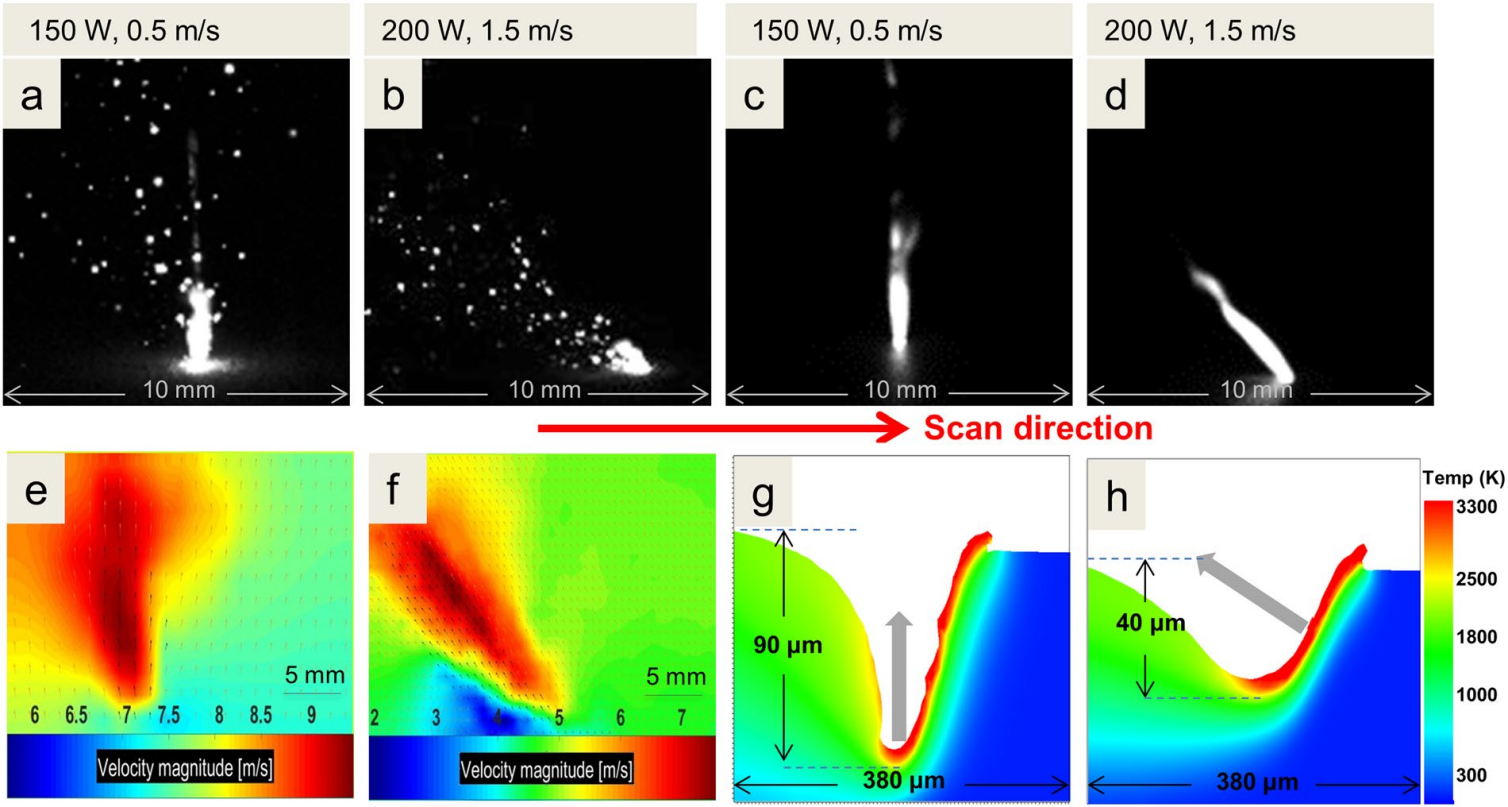
Spatter ejection associated with a SS316L powder layer (**a**,**b**) and for a SS316L plate (**c**,**d**) for two parameter cases: 150 W, 0.5 m/s and 200 W, 1.5 m/s. The camera frame rate was 100 kfps with an exposure time of 8 μs. Laser scanning is performed from left to right. Both cases are in an Ar environment. (**e**,**f**) Particle Image Velocimetry (PIV) was used to quantitatively measure projected velocities. Average velocities of >9 m/s are observed at the center of the plume for the 150 W, 0.5 m/s case and >7 m/s for the 200 W, 1.5 m/s case. Black arrows show actual particle path data and orange arrows are interpolated data. (**g**,**h**) Simulations showing regions above boiling in the melt pool depression for a bare plate. The arrows indicate the expected direction of the vapor plume.

Figure 1a,b shows a macroscopic view of spatter generated from SS316L powder when irradiated with two processing parameter sets: (a) laser power P = 150 W and scan speed u = 0.5 m/s, and (b) P = 200 W and u = 1.5 m/s (see Methods for additional details). The camera is placed approximately 90° to both the laser processing beam and scan path, collecting images at 100,000 frames per second (100 kfps) as the laser is scanned horizontally left to right. Both experiments were performed at 760 Torr of Ar gas. Figure 1a shows a column of droplets rising nearly vertically from the melt pool while Fig. 1b shows a similar amount of droplets ejecting at approximately 47° from the vertical. In contrast, for the bare plate shown on Fig. 1c,d under the same processing conditions, droplets are not visible in the frame shown and only a large plume is observed. We hypothesize that the smaller plume for the powder case is related to plume energy loss to the stream of particles traveling through the plume as we discuss in more detail below. In a sequence of 2000 frames sampled over 20 ms, no droplet ejection was observed for the bare plate, while ~20 droplets/ms were observed for the powder layer sample under the same camera exposure time. Videos corresponding to Fig. 1a–d are provided in Supplementary Movie 1. These results illustrate the importance of the powder in the PBFAM spatter generation process and point to a particle motion mechanism as described later.

### Ejection velocity and ejection angle

The projected ejection velocity magnitude was characterized using particle image velocimetry (PIV) (see Methods for details). Figure 1e,f shows the velocity profile averaged from a subset consisting of 120 image pairs (240 total frames). For both parameter cases, the velocity is highest at the center of the plume and decreases away from the center. At P = 150 W, u = 0.5 m/s, the maximum velocity is 9.8 m/s, and the mean velocity is 8.3 m/s. The mean velocity has a small horizontal component of 1 m/s and a dominant vertical component of 8.2 m/s. At 200 W, 1.5 m/s, the maximum velocity is 7.8 m/s and the mean velocity is 6.3 m/s. The velocity components are distributed more evenly with a horizontal component of 4.5 m/s and a vertical component of 4.4 m/s which gives the angle a tilt.

There is considerable difference between the ejection angles for the two processing parameter cases (see also Supplementary Figure 1). The ejection angle depends on the geometry of the melt pool, which is largely governed by the laser power and scan speed. The melt pool vapor pressure change will scale with the peak temperature rise at the melt pool surface which can be approximated as ${\rm{\Delta }}T\propto P/\sqrt{u}$ ^33^. Thus, with increasing P or decreasing u, a higher vapor pressure is expected. To explain the difference in ejection patterns in more detail, a finite element simulation was performed (Fig. 1g,h) with the same experimental laser parameters. When the laser intensity is high enough to evaporate the metal, a depression region forms under the laser spot due to the recoil pressure. A metal vapor plume is emitted normal to the boiling surface regions located at the melt pool bottom and just forward of the bottom along the scan path. For P = 150 W and u = 0.5 m/s, the laser heating is high at this slow scan speed and causes a deep, vertical depression (i.e., keyhole) (Fig. 1g). The vapor restricted by the keyhole walls escapes mostly in the vertical direction, consistent with experimental data in Fig. 1a. At P = 200 W and u = 1.5 m/s, the recoil pressure is lower and not sufficient to form a deep keyhole, but high enough to form a depression in the melt pool (Fig. 1h). The high temperature region at the depression is thinner than the previous case, and spreads out mostly toward the front of the depression. The vapor plume tends to face backwards, in the direction normal to the depression. The maximum temperature achieved is limited by the boiling temperature of the liquid metal (for a given ambient pressure). By comparing the two simulation cases, there is more liquid melt with temperatures above 2000 K in Fig. 1g than is shown in Fig. 1h, and also the length of the boiling surface in Fig. 1g, which defines a deeper melt pool, is greater than that in Fig. 1h. The increase in the inclination angle as the scan speed becomes higher is also observed in welding studies^34^.

### Droplet ejection from a bare plate

**Figure 2:**
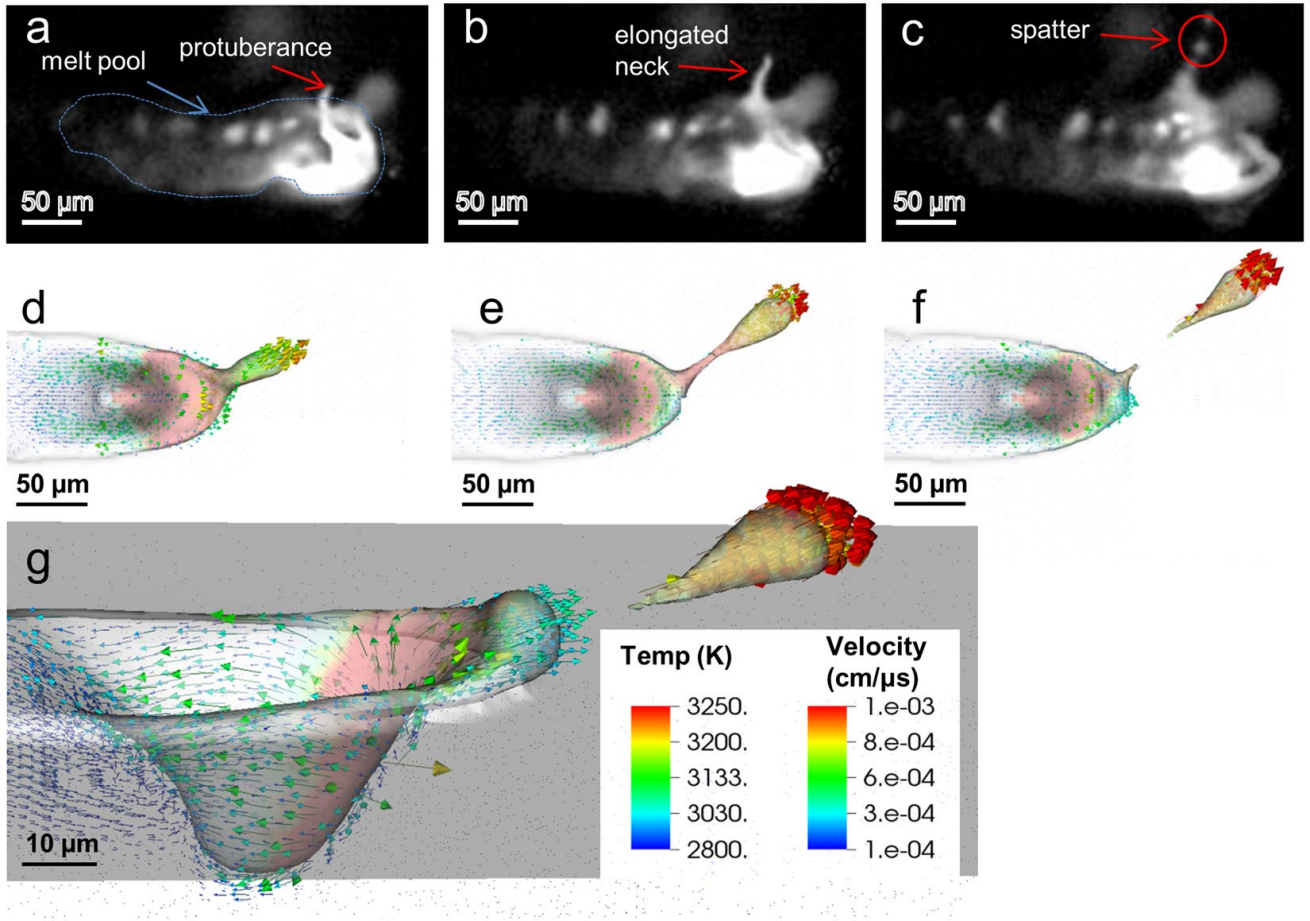
Comparison between experiment (**a**–**c**) and simulation (**d**–**g**) of the laser-driven spatter process for a bare SS316L plate, showing good qualitative agreement. The experiment consists of three experimental snapshots recorded at 1 Mfps in the top region which shows droplet formation and ejection. In (**a**–**c**), the illumination laser is off resulting in self-illumination of the melt pool and surrounding region through incandescence. The spatter forms as a protuberance in (**a**), elongated neck in (**b**) and escapes as a spherical droplet in (**c**). Experimental parameters: P = 600 W, u = 3.0 m/s. (**d**–**f**) Simulation of three snapshots that represent the experimental data. (**g**) Semi-transparent side view of the simulation in (**f**) showing the depression at the front of the melt pool with complex liquid flow and droplet ejection angle relative to the surface of the plate. Simulation parameters: P = 200 W, u = 1.5 m/s.

In order to understand the formation of droplet ejection from the melt pool region, a series of experiments coupled with simulations of increasing physics complexity were performed. Figure 2a–c is composed of experimental snapshots derived from frames of high speed video at 1 million frames per second (Mfps) showing a droplet ejection due to recoil pressure from a SS316L bare plate melt pool (see Supplementary Movie 2). The laser parameters used here is P = 600 W and u = 3.0 m/s. The background is dark due to the illumination laser being off while the melt pool appears bright from incandescence: (a) at the front of the melt pool, a protuberance can be observed, (b) the connection between the melt pool and the elongated ‘necked’ portion thins out, and finally (c) a droplet of diameter ~8 μm breaks away at 25° clockwise from the center of the depression, and travels at approximately 18 m/s. This particular spatter generation event is completed in 2 μs.

To explain the experimental observation of droplet ejection from the melt pool, we begin with a simulation of a SS316L bare plate irradiated with a laser beam normal to the surface. The powder is first excluded in the simulation in order to dissociate the effect of the powder from that of the plate. The simulation parameters used are P = 200 W and u = 1.5 m/s. The middle row of Fig. 2 shows simulation results that describe the spatter forming as a significant topological change that originates at the rim of the depression under the laser spot. The red region corresponds to temperatures close to the boiling point ~3300 K. Figure 2d shows a large droplet forming at 45° clockwise at the front of the melt pool; the melt connection thins out in Fig. 2e, and escapes in Fig. 2f. The side view in Fig. 2g shows the relative location of the spatter, about 25° from the surface of the plate. The simulation agrees well with the experimental data in reproducing the elements of droplet formation and ejection mechanisms associated with melt pool breakup effects. Although the simulation parameters used here differ from the experimental parameters, we note that very similar ejection patterns are experimentally observed across different power and scan speed.

We note that a complex and strong dynamical flow is characteristic of the high temperature liquid melt subjected to high thermal gradients. The simulations in Fig. 2d–f show the liquid flowing vertically mainly at the front of the depression, as indicated by the velocity vector field, but possessing a rotational component along the sides (Fig. 2g). Spatter forms when the liquid at the rim of the depression acquires enough kinetic energy to escape the pull of the surface tension. The liquid motion is directly related to the strength of the vapor recoil force which depends on how much laser energy is being deposited.

For molten micro-droplets to escape, the kinetic energy (dynamic pressure) of the melt must exceed the capillary pressure, which can be expressed as^22^:

1 $${\rho }_{L}{u}^{2} > \frac{\alpha }{R}$$

where α is the surface tension, *ρ*_L_ is the liquid metal mass density and *R* is the melt radius of curvature. For *R* = 200 μm and *α* = 1.5 N/m, the escape velocity for a SS316L melt pool is found to be approximately 1 m/s, in agreement with estimates by Kaplan *et al*.^22^. With the increase of *P* or reduction in *u*, the temperature and recoil pressure also increase, which leads to a deeper depression. He *et al*. demonstrated for the case of laser welding that doubling the laser power can increase the ejected particle diameter and broaden the size distribution. We note that the temperature rise also reduces the surface tension and reduces the required kinetic energy for droplet breakup per Equation (1). However, from Equation (1), ejection of droplets smaller than 8 μm is typically not expected since this would require the melt flow to travel in excess of 5 m/s which is somewhat larger than that observed from our measurements (about 1–3 m/s).

### Simulation of droplet ejection involving a powder layer

**Figure 3:**
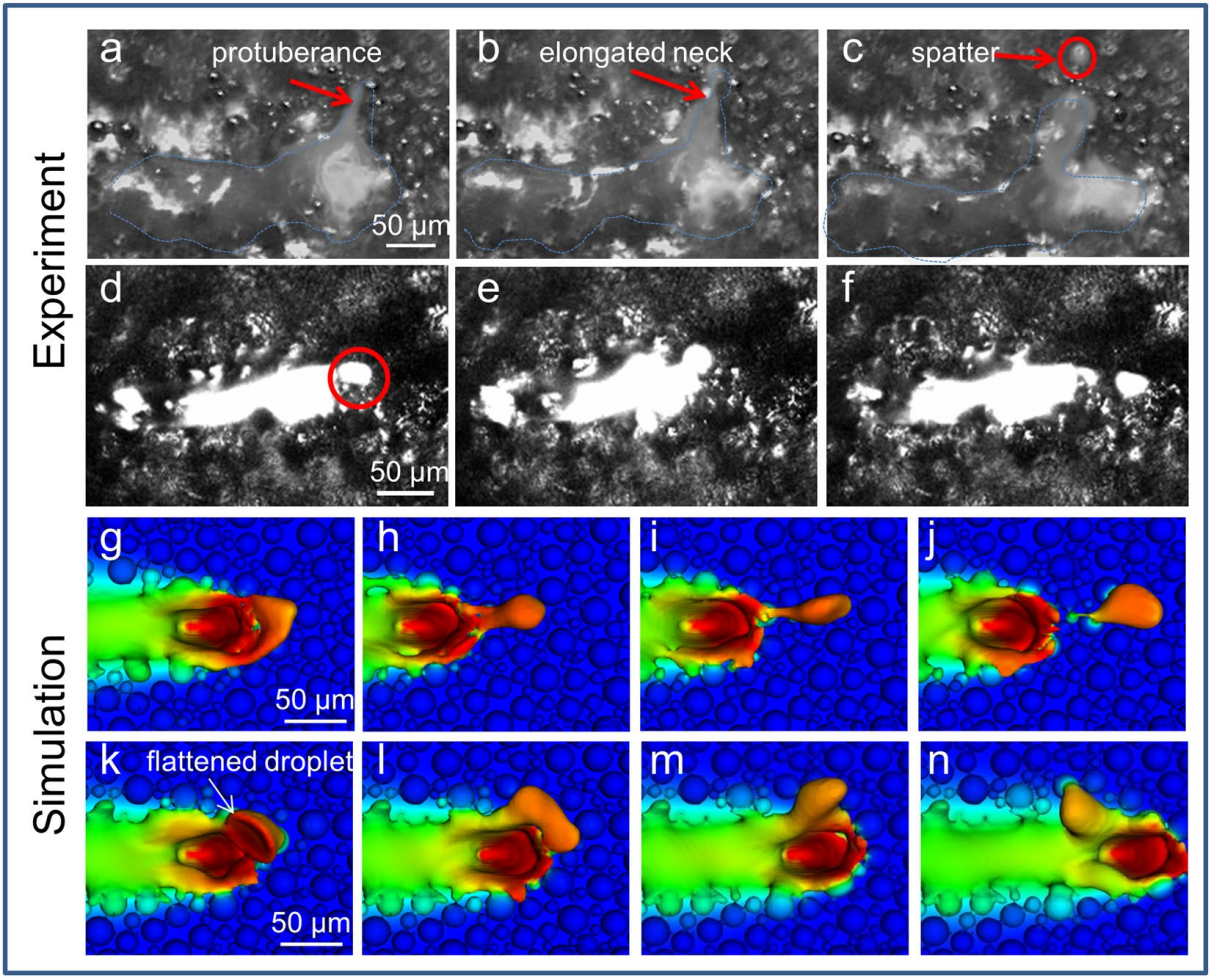
Comparison between experiment (**a**–**f**) and simulation (**g**–**n**) of the laser-driven spatter process in powder, showing good qualitative agreement. The experiment consists of experimental snapshots showing droplet formation and ejection. The illumination laser (Cavilux with filter in **a**–**c**; Thorlabs, no filter in **d**–**f**) is on and the powder can be observed in the background. (**a**–**c**) illustrates a small 10 μm droplet forming and ejecting away from the melt similar to a bare plate. The melt pool is highlighted with blue dashed lines. The spatter forms as a protuberance in (**a**), elongated neck thins out in (**b**) and escapes as a spherical droplet in (**c**) (circled in red). (**d**–**f**) illustrates a larger droplet forming from liquid buildup at the front of the melt due to the presence of powder (circled in red). The melt collects in front of the depression in (**d**) and is plowed forward in (**e**) while collecting mass and escapes the melt pool in (**f**) to land a short distance. The simulation (**g**–**j**) shows four snapshots representing the droplet ejection in (**d**–**f**). Snapshots (**k**–**n**) shows a sub-threshold ejection event. The droplet is flattened on the left side in (**k**), is entrained by the rotation flow towards the back and subsumed by the melt pool (**l**–**n**). Experimental parameters Ti6-4, P = 300 W and u = 1. 5 m/s for (**a**–**c**) and SS316L, P = 200 W, u = 2.0 m/s for (**d**–**f**) and simulation parameters SS316L, P = 200 W, u = 1.5 m/s.

The addition of a powder layer to a bare plate adds more complexity to the melt pool dynamics and affects the forward flow motion (Fig. 3). In Fig. 3a–c and Supplementary Movie 3, the illumination laser is on and powder in the background is visible. A protuberance forms at the rim of the depression in (a), the elongated neck thins out in (b), and a spherical droplet is ejected in (c), similar to the ejection behavior from a bare plate. The presence of powder hinders the forward flow motion since the recoil momentum has a small component along the beam scanning direction (with a pressure of ~7.5 kPa close to boiling), leading to a buildup of liquid molten material ahead of the laser beam. Ejections from forward plowing liquid buildup are typically very large ~25–100 μm. Figure 3d–f and Supplementary Movie 4 shows a large, 30 μm spherical droplet ejected in front of the melt pool with a velocity of ~5 m/s. Also visible in Supplementary Movies 3 and 4 are entrained particles which are swept backwards, a process we will describe in more detail below.

As laser scan speed increases, the deposited energy per unit volume decreases and the depression becomes shallower. The incline angle with the beam direction decreases, hence the recoil pressure’s component along the beam direction increases (as sin (α), where α is the angle between the front incline and the scanning direction), which enhances the horizontal component of the spatter velocity. Eventually, the amount of spatter is expected to plateau and then disappear, since the deposited laser energy will be insufficient to boil the liquid and create an effective recoil force.

For the simulation in Fig. 3g, the droplet forms at the rim of the depression with recoil forces pushing the molten droplet forward. The droplet size increases in Fig. 3h,i as its volume grows due to the addition of cooler liquid from recently melted powder. In Fig. 3j, the spatter has already acquired enough forward momentum that it escapes and lands a short distance ahead.

Through both experiments and simulations, we observe sub-threshold ejection events where protrusions from the melt pool do not completely eject and are instead absorbed back into the melt pool. The action of the recoil vapor pressure of the liquid is evident as the laser causes heating to boiling temperatures with peak simulated temperatures above 3000 K. The radial pressure violently flattens one side of the droplet relative to the laser spot center, as indicated by the arrow in Fig. 3k. The nascent droplet is entrained by the strong rotational melt flow in Fig. 3l,m, and it is swept toward the back where it merges into the melt pool in Fig. 3n. Spatter can still escape in the backward direction if the kinetic energy is greater than the surface tension.

### Entrainment-driven spatter

From high speed movies (Supplementary Movies 3 and 4), in addition to observing liquid droplets ejecting directly from the melt (Figs 2 and 3), a strikingly different behavior is also observed. As the laser is scanned across the powder bed, many particles are observed being swept away, upward and rearward relative to the scanning direction. The motion of these particles is due to vapor driven entrainment^18^ and this effect is found to be an even more dominant mechanism for spatter formation than laser induced recoil pressure. The observation of entrainment-driven spatter is the key finding of this study, and is described in detail below.

Currently, it is computationally challenging and expensive for our simulation code to adequately treat multi-phase flow, fluid-structure interactions and contact mechanics involved in laser-vapor-driven particle entrainment. Particle entrainment by gas flow on granular bed has been studied before^35^ however the main difference here is that the jet size is comparable to the particle size, which allows us to observe entrainment of single particles and not ensemble averages. In lieu of finite element simulations to rationalize the experimental results, we will turn to basic physics formalisms to guide us. Initially the powder particles are at rest in the powder layer prior to the vapor flow field associated with the melt pool coming in contact with them. Due to the requirement that powder have good ‘flowability’ in powder bed fusion systems, the powder particles are easily detached from the surface and each other. The drag force that leads to detachment and eventual entrainment is given by the Stokes formula^36^:

2 $${\rho }_{p}\frac{4}{3}\pi {a}^{3}\frac{dv}{dt}=6({v}_{g}-{v}_{p})\pi \eta a$$

where *v*_*p*_ is the particle velocity, *v*_*g*_ is the gas velocity, *ρ*_*p*_ is the density of a solid particle with radius *a* and gas viscosity *η*. The solution of this equation with *v*_*p*_ = 0 at *t* = 0 is given by expression,

3 $${v}_{p}={v}_{g}(1-{e}^{-\frac{t}{\tau }});\,\tau =\frac{2{\rho }_{p}{a}^{2}}{9\eta }$$

where *τ* is the entrainment time. For stainless steel particles (*ρ*_*p*_ ~ 8000 *kg/m* ^3^), the entrainment time in ms is simplified as *τ* = ba^2^. At T = 3000 K corresponding to the vapor temperature, *η* ≈ 6.9 × 10^−4^ g/cm-s and b = 25 ms/μm^2^ such that for *a* = 10 μm, *τ* ≈ 2.5 ms. Assuming the vapor jet is comparable with the laser beam diameter, the particle dwell time in the jet is t = d/u = 50 µs for a D4σ Gaussian laser beam diameter of d = 50 µm and scan speed u = 1 m/s. Given *t* ≪ *τ*, equation (3) can be estimated by ${v}_{p}\approx {v}_{g}\frac{t}{\tau }$ and the particle velocity increases linearly with time.

From Supplementary Movies 3 and 4, particles are affected by the wake of the jet stream 3–4 beam diameters away so the effective entrainment distance extends to 150–200 µm. The vapor jet velocity from our simulations for SS316L is up to 700 m/s for the case of P = 225 W and u = 1.4 m/s^18^. The entraining gas velocity and temperature field magnitudes gradually decreases with increasing distance from the jet center. For particles with radius *a* = 10 µm and a vapor temperature of 3000 K, the maximal entrainment velocity is *v*_*p*_ ≈ 0.06 *v*_*g*_ and for a gas velocity of a few hundreds m/s, the particle velocities can be in excess of 10 m/s. Furthermore, the entrainment velocity is inversely proportional to particle density such that for less dense materials such as titanium, higher velocities and more spatter is expected as compared with stainless steel. Experimental comparisons of spatter ejections associated with these two material systems are shown below, which support this conclusion.

**Figure 4:**
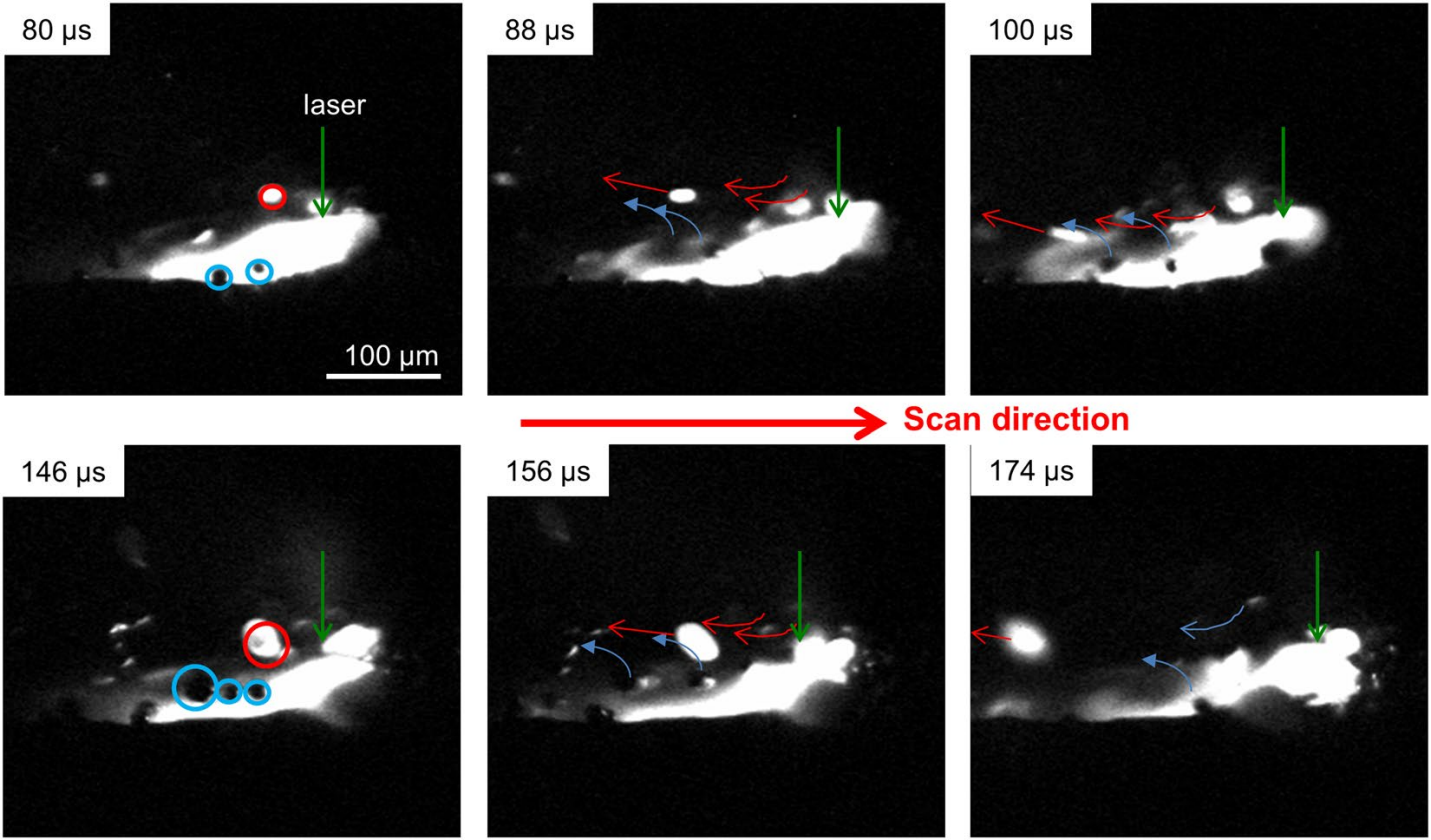
High resolution imaging of the melt pool formation at 500 kfps. To more clearly observe the rapid heating and incandescence of the entrained particles, the external illumination source is turned off. The laser power for the time sequence shown is P = 300 W and u = 1.5 m/s. The series of images show the time progression from 80 µs to 174 µs. As the laser is scanned from left to right, particles are observed being swept up and backward with the arrow denoting the movement. The first image at 80 µs shows the location of the laser spot (green arrow) at the front of the melt pool. Hot particles are white in the image (circled in red) and cold particles are black (circled in blue). At 88 µs, the cold particles are lifted up and swept in a motion depicted by the blue arrows. Similarly hot particles that exit the laser beam is swept in a motion depicted by the red arrows. At 146 µs, new cold and hot particles are being entrained and the process repeats.

Figure 4 and Supplementary Movie 5 shows a sequence of this entrainment process over time for a track irradiated at P = 300 W, u = 1.5 m/s. The movie is shown with the illumination laser off to best capture two distinct types of moving particles, dark and bright, in the images. At 80 μs, the particles circled in blue in Fig. 4 rise from the powder bed with an initial velocity of about 1 m/s and once entrained by the gas, are swept opposite to the laser motion and upward with a velocity of ~4 m/s. Note that these particles, displaying negligible incandescence, do not interact with the laser. The incandescent, hot particles at 80 μs (circled in red in Fig. 4) are initially dark but become entrained by the gas flow, and pass through the laser path and are rapidly heated to emit bright incandescence. The dark particles initially have a velocity of 0.5–1 m/s but increase their speed to about 4 m/s once fully entrained at a height level of ~20 μm from the plate surface. Once the dark particles cross the laser path, they are irradiated and rapidly turn into bright particles in less than 10 μs while their velocities increase to 6–15 m/s with maximum values observed near 20 m/s. The sudden increase in velocity over a short 100 μs time span corresponds to an acceleration of greater than >10^6^ m/s^2^. Not all entrained particles are ejected – in fact, a portion are subsumed into the melt pool and contribute to the PBFAM deposition process (it should be noted that, prior to this study, the contribution of entrained particles to the formation of melt tracks in PBFAM was completely unknown and unexpected). Based on analysis of 120 videos with power ranging from 200–300 W and scan speed from 1.5–2.0 m/s, we concluded that 60% of the total spatter generated is from hot ejections, 25% from cold ejections, and 15% from recoil pressure induced ejections. From these measurements we thus conclude that the spatter observed in Fig. 1 are mostly hot ejections from entrainment. While the conditions and materials vary among commercial PBFAM systems, we propose here that the vast majority of what is observed as spatter is also mostly generated by the entrainment process described here.

### Mechanism involved in the entrainment of particles

**Figure 5:**
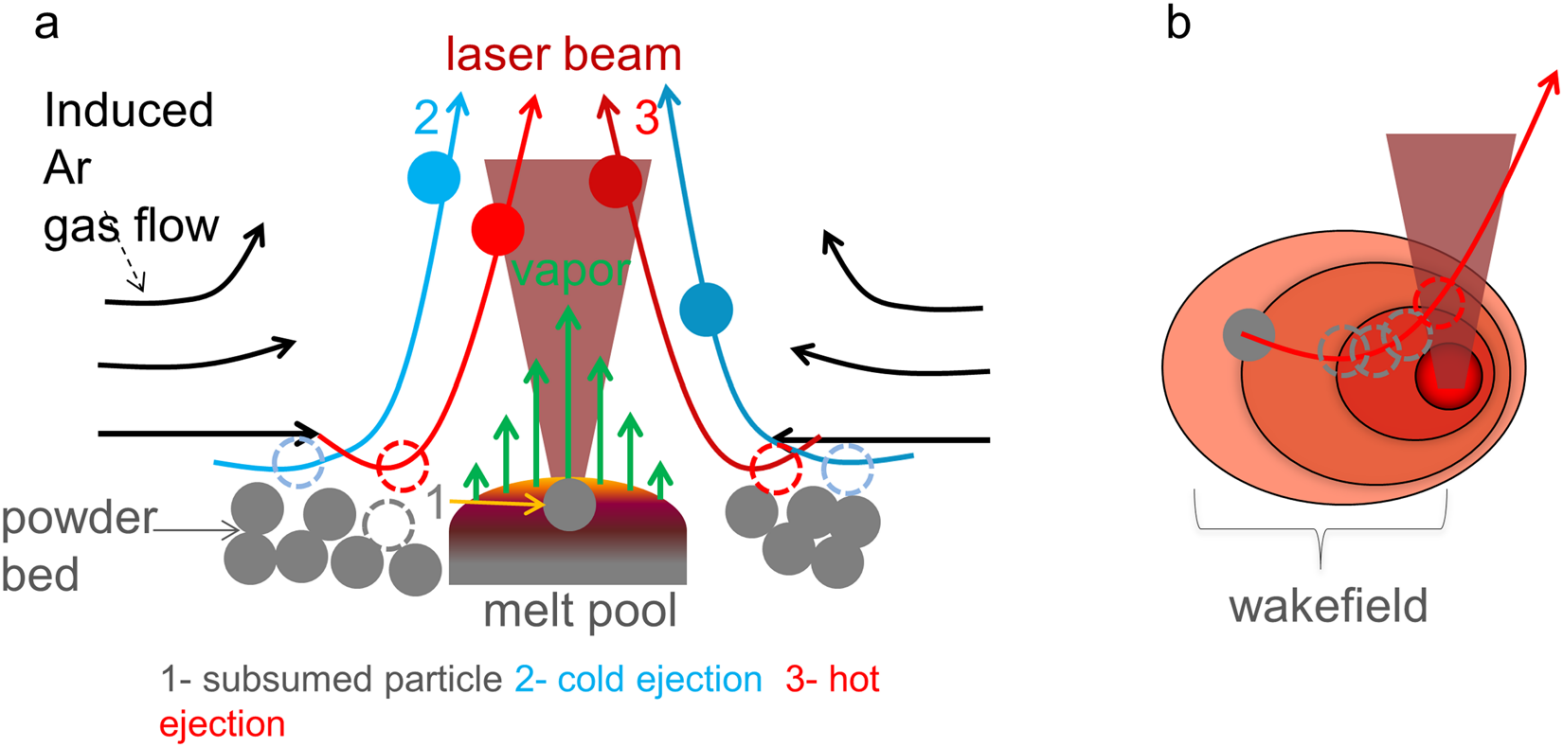
(**a**) Schematic depicting particle entrainment of the powder bed by an induced argon gas flow for a stationary laser beam. A vapor jet creates a low pressure zone which leads to an inward gas flow that entrains particles and leads to three distinct trajectories: particles with low vertical momentum are swept into the melt pool and subsumed (1), particles with higher vertical momentum but originating >2 melt pool widths away are swept into the trailing portion of the vapor jet and ejected as cold particles (3) and particles with roughly the same vertical momentum as (2) but originating closer to the point of laser irradiation (<2 melt pool widths) are swept into or near the laser beam itself rapidly heat and are ejected as incandescent, hot particles. (**b**) For a moving laser beam, a non-uniform jet stream wakefield is induced and extends 3–4 beam diameters away.

Figure 5 summarizes the interaction of the powder particles with the evaporation-driven Ar gas flow. Figure 5a depicts the case for a stationary beam (or similarly a scanned beam viewed head-on). The metal vapor flux from the melt pool induces an inward gas flow that pulls the particle along the vapor flow direction, similar to the physics of a submerged jet described in ref. 37. The entrained particles are accelerated by the gas flow resulting from the vapor jet toward the melt pool and depending on the local trajectories will be subject to at least three main outcomes: (1) pulled into the melt pool and become subsumed, (2) travel toward the vapor jet but miss the laser beam and eject as cold particles or (3) travel toward the vapor jet, intersect the laser beam leading to rapid heating and ultimate ejection as hot, bright particles that are observed on the macroscopic scale as molten spatter or sparks. Entrainment for a stationary beam is shown in Supplementary Movie 6. We note that the effect of a vertically-oriented metal vapor jet causing a low pressure region and pulling in the surrounding gas is a common phenomenon akin to the situation of a firestorm at the macroscale. The heated air entrains ember and soot particles upward toward the cooler atmosphere and the influx of surrounding air provides both oxygen and entrained material for combustion^2^.

For a moving beam, the onset of the gas flow around the vapor jet takes some time and hence the laser beam motion establishes a flow field elongated along the scan direction (Fig. 5b). Particles initially attached to the surface through van der Waals forces are detached from the powder bed and accelerated mainly in the wake of the jet stream that extends toward the rear of the melt pool.

Since the thermal conduction time *τ*_*C*_ = *a* ^*2*^ */4D* ~ 25 μs across a steel particle is much longer than the heating time of 6 µs (Supplementary Equation S1), there are conditions under which only a portion of the particle is in the boiling state. Partial illumination on the side of a particle can lead to it being “side swiped” back into the melt pool where it is subsumed (Supplementary Movie 7).

Particle–particle collisions will also have an effect on the particle dynamics. A large transfer of momentum occurs between two colliding particles and the motion of each changes abruptly (Supplementary Movie 7). For collisions between two hot droplets, the particles may merge to form a large droplet (Supplementary Movie 8). This merging process and the resulting large particle formed after cooling can potentially lead to lack-of-fusion defects in subsequent build layers in a PBFAM process^38^.

Hot particles can also undergo very fast acceleration due to the spatial non-uniformity of the laser irradiation. From Supplementary Equation S2, for *p* = 300 kPa and for *a* = 10 µm this corresponds to an acceleration of 3 × 10^6^ m/s^2^, which is consistent with estimates derived from the experimental results of Fig. 4 and Supplementary Movie 5.

Once outside the laser beam, the spatter droplets move through the surrounding Ar gas and cool down. The Reynolds number *Re* = *ρvp a/η* is nearly 1, and the flow around the ejected droplet is laminar. In vacuum, a particle ejected upward with velocity *v* = 1 m/s drops back in 2 *v/g* seconds ~200 ms (*g* is the gravitational constant). In Ar, the drag forces decrease the height that the particle reaches but do not change the flight time appreciably. The time *t*_*c*_ ≈ 13 ms (Supplementary Equation S3) is much shorter than the flight time and most of the droplets fall down as solid spherical particles, preserving the shape developed during flight.

**Figure 6:**
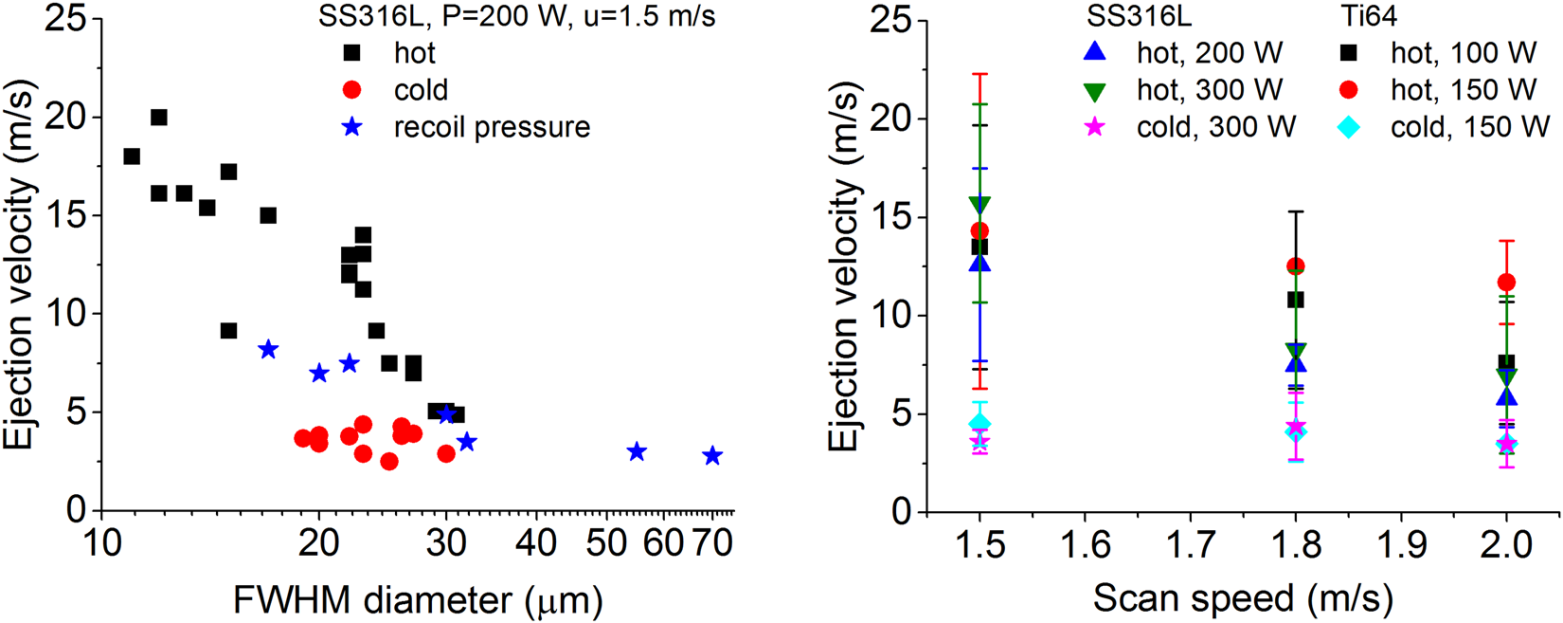
(**a**) Velocity-size distribution for SS316L at P = 200 W, u = 1.5 m/s. Hot-entrained ejections (black squares) had projected velocities ranging from ~6 m/s for 30 µm particles to ~20 m/s for 10 µm particles. Cold-entrained ejections (red circles) had projected velocities ranging from 2 to 4 m/s. Melt pool ejections (from recoil pressure and not involving an entrainment effect) had projected velocities ranging from 3 to 8 m/s. (**b**) Velocity as function of scan speed for SS316L and Ti64 at different laser powers.

To further analyze the spatter characteristics, we tracked the ejected motion frame-by-frame using MtrackJ (see Methods for details). As previously noted, it is not possible to resolve the angular trajectories completely using one camera, and therefore the velocities calculated here are stereographic projections of velocities onto the plane perpendicular to the camera line of sight. Figure 6a shows a velocity-size distribution for SS316L at P = 200 W and u = 1.5 m/s. The hot particle ejections due to entrainment had projected velocities in the range 6–20 m/s dependent on particle size which is determined by taking 1D intensity line-out profile and fitted to a FWHM Gaussian diameter. The hot particle velocities are consistent with the PIV data in Fig. 1e,f. For cold particle ejections due to entrainment, the velocities range from 2–4 m/s and do not appear to vary much with particle size. Melt pool ejections from vapor recoil pressure (and not involving an entrainment effect) are 3–8 m/s inversely dependent on the size. Figure 6b displays velocities for SS316L and Ti64 as function of the scan speed for different powers. The velocities for Ti64 were observed to be higher than SS316L at the same scan speed due to the lower density as predicted by Equation (3).

While the velocity-size data corresponding to each type nearly crosses at ~30 μm, the dependence over diameter shows different behavior for the three types of spatter. In addition, although recoil pressure driven droplets only account for a small portion of the total spatter, the diameters of the particles are larger with a broader size distribution. The largest diameter particle is likely the most important source of contamination since melting of a subsequent layer may not melt spattered particles with diameter greater than the layer thickness. Ideally the spatter ejection would be high velocity and at most in the size range of the powder bed particles themselves, to avoid pore defect formation and allow ejected spatter to be carried far away from the build area. Entrained particles typically have sizes in the range of powder bed particle sizes, however, there are cases where the particles are larger (one example is two hot particles merging together as shown in Supplementary Movie 8).

While it is also important to know the temperature profiles of the various types of ejected particles, it is difficult to accurately quantify them since the recoil pressure driven droplets and hot, entrained particles are both in the boiling state >3000 K. However, the surface temperatures associated with the different ejection processes can be different, and is worth investigating in future studies, particularly one in which we have a full, two-phase flow simulation to accompany the high speed observations.

**Figure 7:**
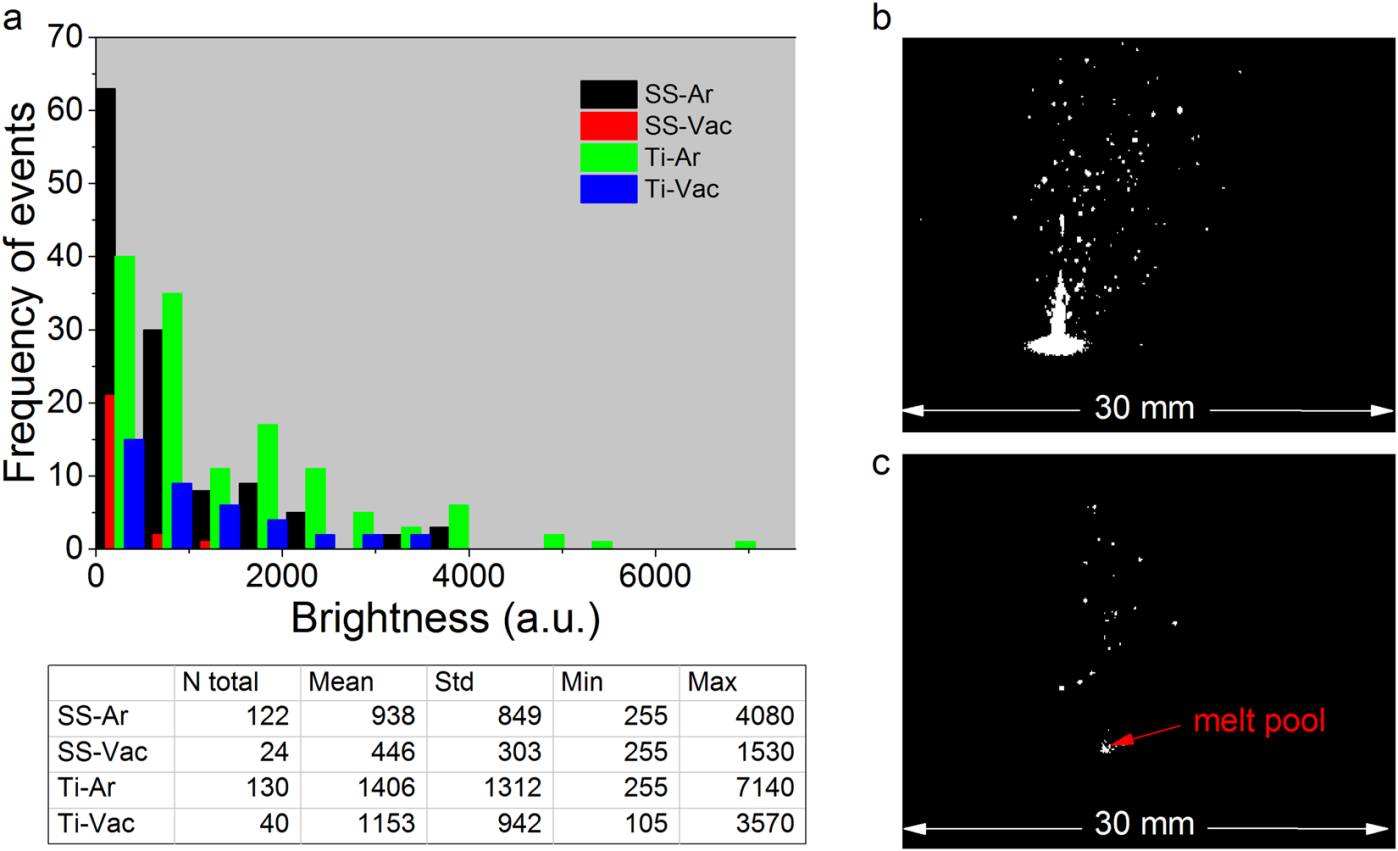
Comparison of spatter ejection for SS316L and Ti64 powder in 10^−3^ Torr Vac and 760 Torr Ar over 5 ms time frame. (**a**) Histogram of brightness which is taken as the sum of the total number photons (“N total”) above the background with bin size = 255. “Mean, Std, Min, Max” on the Table, indicates the mean, standard deviation, minimum and maximum brightness. For SS316L, in Ar, there are 5 × more ejections that appear with 2 × higher intensity. Ti64 had more ejections than SS316L in Ar and vacuum. Experimental images recorded for SS316L and shown in (**b**) argon and (**c**) vacuum were taken at a camera frame rate of 100 kfps and an exposure time of 8 μs. Laser power P = 200 W, scan speed u = 1.5 m/s.

As a consistency test to the entrainment mechanism, in Fig. 7 we compared the ejection rates for a powder substrate in near vacuum and Ar gas at a fixed camera exposure time of 8 μs. Based on brightness level for the ejected particles, there is a noticeable decrease in spattering at 10^−3^ Torr (Fig. 7c) vs 760 Torr (Fig. 7b) for SS316L. Figure 7a compares the total number of ejections for SS316L and Ti64 over a 5 ms time span. For SS316L, the total ejections drop from 122 at 760 Torr Ar to 24 at 10^−3^ Torr, with a 2 × drop in brightness. It is not clear if the decrease in brightness is incidental or due to pressure conditions. Comparing SS316L to Ti64, spatter generation from titanium are 1.5 × brighter. The heat capacity of Ti64 is almost two times smaller than that for SS316L, and for the same *P* and *u* we expect a higher melt pool temperature and more intensive spatter generation. Although more related to recoil pressure driven spatter, the general trend is consistent with previous studies of laser welding which show that spatter is generated more easily for titanium than stainless steel^39^.

However, what appears puzzling is that spatter generation is still observed even in the near absence of ambient gas flow entrainment. Although we have no clear answer for this (due to the fact we could not record high resolution images at low pressure), we propose a few explanations. From collisions, some particles can be pushed into the vapor jet and accelerated into the laser beam generating spatter, an effect that has been observed previously which shows particle colliding with the surrounding powder to form a denudation zone^18^. Particles in the path of the laser can also become spatter when heated. Another explanation is that residual air trapped between particles, or gas desorbed from the metal surface, can be heated up and expand which pushes particles upward into the laser beam^40^. Further experiments are needed to validate these hypotheses.

The loss of material from the track forming region associated with spatter generation can be important for practical reasons. For the case of recycled powder, knowing the relative amount of material in the powder bed that is a result of spatter could help predict the limits of recycling since spattered particles can affect the powder size distribution and/or composition which can prevent effective spreading and/or produce precipitated inclusions.

The mass per unit length of material solidified in building one track is approximately *w*_*t*_ *×* *h* × *ρ*_*solid*_ where *w*_*t*_ and *h* are the width and height of the track respectively, and *ρ*_*solid*_ is the density of the solidified material. Not all particles are used to form the track – some are ejected away and land elsewhere on the powder bed. The total mass per unit length of powder *involved* during the track formation is given by *w*_*d*_ × *l* × *ρ*_*pow*_ for a layer thickness *l* (*~*60 µm), powder density *ρ*_*pow*_ (~0.5*ρ*_*solid*_ assuming a 50% porosity), and width of the denudation zone *w*_*d*_. For Ti64 at P = 75 W and u = 0.5 m/s, the dimensions of the solidified track were measured to give *h* ~ 60 µm, *w*_*t*_ ~ 150 µm, and *w*_*d*_ ~ 350 µm^18^. The percent of material used in building a single track (*w*_*t*_ *hρ*_*solid*_ */w*_*d*_ *lρ*_*pow*_) is approximately 85% while the remaining 15% are particles that are redistributed across the powder bed and get redeposited. The calculations here are based on one parameter case as an example and are expected to vary with processing conditions. We also note that in an actual build, the first track will have the most surrounding powder as compared with subsequent adjacent layers.

## Conclusion

In conclusion, we have presented experimental and simulation results which have elucidated the physics of the spatter generation and role of micro-particle entrainment in PBFAM processes. Simulations of melt pool ejections agree well with high speed video of liquid droplet ejections from laser induced recoil pressure. However, we have discovered a more dominant process related to metal vapor-driven particle entrainment that leads to more efficient material ejection for PBFAM processes. Experiments showing the relatively large spatter generation rates associated with powder layers as compared with bare plates under the same laser parameters support this conclusion. The entrainment process leads to both local denudation and the distribution of molten droplets across the powder bed, each potential sources of defects in PBFAM. We conclude that 60% are hot entrainment ejections with velocities of 6–20 m/s, 25% are cold entrainment ejections with velocities of 2–4 m/s, and 15% are recoil pressure induced droplet breakup ejections with velocities of 3–8 m/s. The results presented here should stimulate further development of new multi-phase flow models to address the detailed physics of laser-vapor-driven particle entrainment in order to better understand process conditions which can lead to PBFAM component defects.

## Methods

### Experimental setup

A 600 W fiber laser (JK lasers, model JK600FL) is directed through a 3-axis galvanometer scanner (Nutfield technologies) and into a 15 × 15 × 15 cm^3^ vacuum chamber through a high purity fused silica window. The ~f/20 optical system results in a focused D4σ diameter of ~50 μm at the sample. The vacuum chamber is evacuated using a turbomolecular pump and purged with Ar. Residual oxygen content is measured using a photoluminescence quenching meter (Ocean Optics Neo Fox) and the concentration of oxygen is below 0.01%, the lower limit of the oxygen sensor’s measurement range. Experiments are performed at 1 × 10^−3^ Torr or 760 Torr Ar. Pressure is controlled using a combination of purge and pumping rates. An ultra-high-speed camera with microscope optics (Mitutoyo 10x/0.28NA, Infinity K2) is placed outside the chamber to image the melt pool. Imaging is performed at up to 100 kfps frames per second (fps) using a Photron SA-X2 or up to 1 Mfps using Shimadzu HPV-2, with an optical resolution of ~5 μm. For illumination of the surface, either a 700 mW, 638 nm CW diode laser (Thorlabs, model L638P700M) or a low coherence 810 nm, pulsed laser with 500 W peak power (Cavitar, model Cavilux HF) are employed. Due to the limited field of view from imaging with a single camera, out of plane components of the velocity could not be determined, such that all velocity measurements are in-plane projections. A 25.4 mm diameter, 3.2 mm thick build plate of the same composition as the powder is used with a bead-blasted and ultrasonic cleaned surface.

Characterization of average particle velocity is performed using PIVlab^41^, an image correlation algorithm written in Matlab. Each image frame is divided into many small interrogation windows, and the particle trajectory is determined from cross correlation of the interrogation windows from a pair of image using fast Fourier transforms. The correlation comprised of 2 passes with the 1^st^ pass comprised of an initial 64 × 64 pixel interrogation area for a coarse velocity calculation, followed by a 32 × 32 smaller interrogation area for finer vector resolution. Tracking of individual particle trajectory is performed using MtrackJ, an ImageJ plugin^42^ by analyzing particle displacement frame by frame. This has the advantage of tracking single particle ejections and their emission angles.

### Materials

Powders used are stainless steel type 316 L (SS316L, CL20ES, Concept Lasers) and Titanium alloy Ti-6Al-4V (Ti64, AM64gr5, Additive Metal Alloys). Bare plate substrates were obtained from McMaster Carr. Wire EDM was used to machine the 1/8″ thick stock into 1″ diameter discs matching the sample holder size. The discs were lightly bead blasted on the side where powder is spread to provide a rough surface to aid in preparing uniform layers of powder. The discs are washed in soap and water before use. Powders were manually applied using a stainless steel razor edge to simulate the spreading process in a production machine. Diameters of the powder particles for SS316L and Ti64 samples were 30 ± 10 μm. Nominal thicknesses for the metal powder layers for all measurements were ~60 μm.

### Data availability statement

The datasets generated during and/or analysed during the current study are available from the corresponding author on reasonable request.

## Electronic supplementary material

Supplementary Information

Supplementary Video 1

Supplementary Video 2

Supplementary Video 3

Supplementary Video 4

Supplementary Video 5

Supplementary Video 6

Supplementary Video 7

Supplementary Video 8
