# Supplementary material for: Metal vapor micro-jet controls material redistribution in laser powder bed fusion additive manufacturing
Source: Sci Rep. 2017 Jun 22;7:4085. doi: 10.1038/s41598-017-04237-z (PMC5481335; doi:10.1038/s41598-017-04237-z)
Supplement: Supplementary file 1 — Supplementary Information [file 41598_2017_4237_MOESM1_ESM.pdf]

Metal vapor micro-jet controls material redistribution in laser powder bed fusion additive manufacturing

Sonny Ly[1], Alexander M. Rubenchik[2], Saad A. Khairallah[3], Gabe Guss[4] and Manyalibo J. Matthews[1*]

[1]Materials Science Division, Physical and Life Sciences Directorate, Lawrence Livermore National Laboratory, 7000 East Avenue, Livermore, California 94550 USA

[2]Laser Science and Systems Engineering, NIF and Photon Sciences Directorate, Lawrence Livermore National Laboratory, 7000 East Avenue, Livermore, California 94550 USA

[3]Computational Engineering, Engineering Directorate, Lawrence Livermore National Laboratory, 7000 East Avenue, Livermore, California 94550 USA

[4]Laser Systems Engineering Operations, Engineering Directorate, Lawrence Livermore National Laboratory, 7000 East Avenue, Livermore, California 94550 USA

*Corresponding Author: Manyalibo J. Matthews
Lawrence Livermore National Laboratory
7000 East Avenue, L-470
Livermore, CA 94550
Email: ibo@llnl.gov
Phone: (+1) 925-424-6762

Supplementary Information

SI-1 Supplementary figure

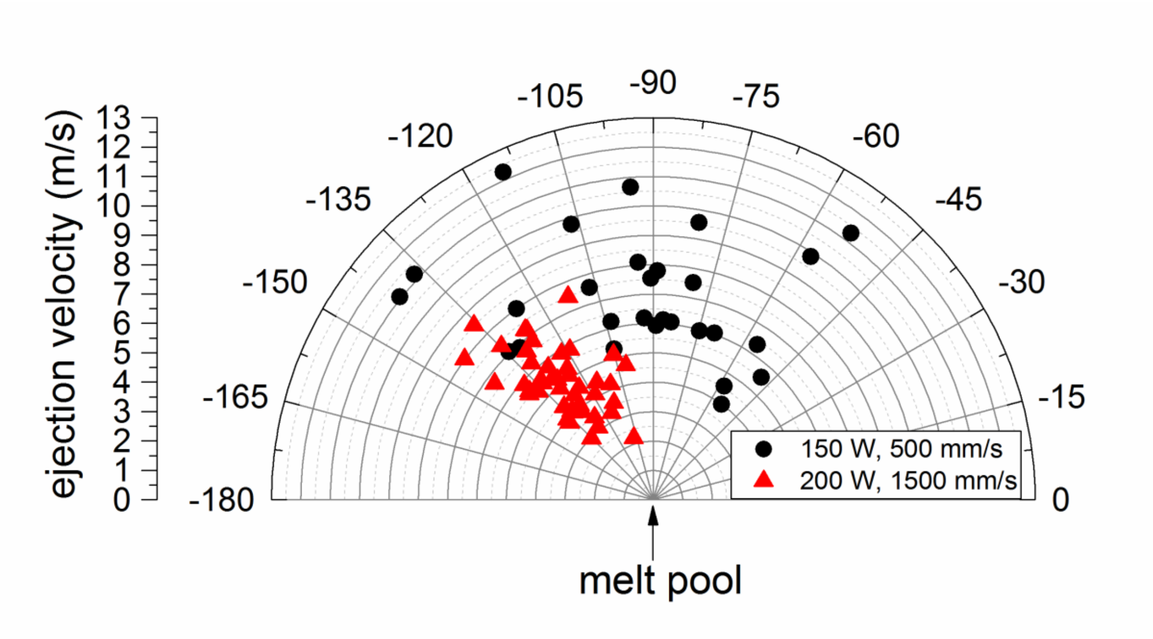

Supplementary Figure S1: Polar plot showing the velocity distribution of spatter for SS316L powder for the two parameter cases 150 W, 500 mm/s and 200 W, 200 mm/s. The data is taken from a subset of a frame in Supplementary Movie 1 corresponding to Figure 1a, b. Each point on the polar plot represents a droplet ejection. The melt pool is shown at the origin.

SI-2 Theoretical framework for the entrainment process

1. Heating of the droplet

Entrained particles that enter the laser beam profile can interact with the laser radiation through direct energy absorption (as compared with most of the powder bed particles which interact with the traveling melt pool and not directly with the laser). The thermal

conduction to the surrounding gas is small (~300 W/cm^2) compared with the energy flux of the laser ~MW/cm^2 and the temperature of isolated particles rapidly increases. Radiative losses from the particle are comparable to thermal losses (~447 W/cm^2 at 3000 K), and are also negligible. We can estimate the particle temperature through the energy balance expression:

$$A\pi a^2 It = \frac{4}{3}\pi a^3 \rho_p C_p T \quad \text{(S1)}$$

where A is the absorptivity, I is the peak laser intensity and C_p is the particle heat capacity. For stainless steel, A = 0.36 [20], ρ_p = 8 g/cm^3, C_p = 0.5 J/cm^2 and equation (S1) can be rewritten in terms of the melting temperature, $T/T_m = t[\mu s] I[\frac{MW}{cm^2}]/\ 36(\frac{a}{10[\mu m]})$. For a typical laser intensity of 10 MW/cm^2, the time to heat up to the boiling temperature is about t = 6 μs.

If we consider the special case where the laser irradiates half of a particle leading to asymmetric evaporative flux,

$$p\pi a^2 = \rho_p \frac{4}{3}\pi a^3 \frac{dv}{dt} \quad \text{(S2)}$$

where p is recoil pressure. At the boiling temperature, the saturated vapor pressure is one atmosphere and since evaporation is a very effective cooling mechanism, the pressure cannot exceed a few atmospheres. From equation (S2) for p = 3 atm and for a = 10 μm this pressure corresponds to an acceleration of 3x10^6 m/s^2.

2. Cooling of the liquid droplets

Once outside the laser beam the spatter droplets move through the surrounding Ar gas and eventually cool down. This cooling can be very efficient due to the forced convection experienced by the particle as it travels. For a ~10 μm, the Reynolds number $Re = \rho v_p a/\eta$

is nearly 1, and the flow around the ejected droplet is laminar. Due to the high thermal conduction of the metal, the particle temperature is treated as uniform. The exact solution of the particle cooling time t_c can be found in[1]:

$$t_c = \frac{1}{3}\frac{\rho_p C_p}{\rho C}\sqrt{\frac{a^3}{D v_p}}. \tag{S3}$$

Here ρ and C are the density and heat capacity respectively of the gas, and D is the gas thermal diffusivity. For Ar at room temperature and pressure, D = 0.2 cm^2/s, C = 0.45 J/(gK), ρ =1.8 x 10^{-3} g/cm^3, and the ratio of the product $\rho_p C_p$ for steel to ρC for Ar is ~0.5x10^4. The cooling time can be written as $t_c[ms] \approx 0.4(a[\mu m]^3/v_p[\frac{m}{s}])^{1/2}$ and for a = 10 µm, $t_c \approx$ 13 ms.

1. Levich, V. *Physicochemical Hydrodynamics*. (Prentice Hall, 1962).

SI-3 Supplementary video descriptions

Supplementary Movie 1: Macroscopic view of incandescent droplet ejections for SS316L powder (top row) vs bare plate (bottom row) for two parameter cases: (left column) 150 W, 500 mm/s, (right column) 200 W, 1500 mm/s. Frame rate 100 kfps, exposure time 8 µs. For all cases, the melt track length is 10 mm and the field of view is 15 mm × 15 mm.

Supplementary Movie 2: Microscopic view of incandescent melt pool and droplet ejections due to recoil pressure for a SS316L bare plate (shown between 6 to 7 seconds in video to correspond with images shown in Fig. 2a-c) SS316L, P = 600 W, u = 3.0 m/s. Frame rate 1 Mfps, exposure time 500 ns, illumination laser off. The field of view is 760 µm × 400 µm.

Supplementary Movie 3: Microscopic view of melt pool motion, particle displacements and droplet ejections from a Ti64 powder layer due to recoil pressure for P = 500 W, u = 0.5 m/s. Droplet ejection can be observed between frames 180 to 225, approximately 12 to 15 second runtime in the video. Frame rate 666 kfps, exposure time 300 ns, illumination laser on with an optical band pass filter blocking most of the melt pool incandescence. The field of view is 500 μm × 360 μm.

Supplementary Movie 4: Large droplet ejection in the forward direction due to molten liquid buildup from melted SS316L powder in the front of the melt pool, observable here between frames 40 and 60, approximation 4-6 seconds of runtime in the video. Processing conditions P = 200 W, u = 2 m/s. Frame rate 500 kfps, exposure time 2 μs, illumination laser on with no optical band pass filter, allowing incandescence from melt pool to be viewed simultaneously with colder surrounding powder layer. The field of view is 500 μm × 416 μm.

Supplementary Movie 5: Particle entrainment and rapid particle heating which leads to incandescent droplets ejecting from the region above the melt pool. Several 'hot' entrainment events can be observed where cold particles enter the region of laser irradiation and become bright prior to going out of focus and ejecting away. Processing conditions P = 300 W, u = 1.5 m/s. Frame rate 500 kfps, exposure time 2 μs, illumination laser off. The field of view is 500 μm × 360 μm.

Supplementary Movie 6: Vapor-driven particle entrainment in a layer of Ti64 powder from a stationary (non-scanning) laser beam with P = 100 W. Frame rate 666 kfps, exposure time 300 ns, illumination laser on with optical bandpass filter to block melt pool incandescence. The field of view is 500 μm × 416 μm.

Supplementary Movie 7: This movie of Ti64 particle dynamics shows two effects: 1) collision between two cold particles (shown around frame 210 or 14 seconds in video runtime) and 2) particles which are "side swiped" by the laser and pushed into the front of the melt pool (example at frame 195 or 13 seconds in video runtime). Processing conditions P = 500 W, u = 1.0 m/s. Frame rate 666 kfps, exposure time 300 ns, illumination laser on with optical bandpass filter to block melt pool incandescence. The field of view is 500 μm × 416 μm.

Supplementary Movie 8: Movie showing the merging of multiple Ti64 particles to form large droplets (see for example between frames 45 and 75 or 4 and 5 second runtime in the video). Large 'spatter' droplets like these can be the source of void defects in PBFAM processing. Processing conditions P = 100 W, u = 0.5 m/s. Frame rate 666 kfps, exposure time 300 ns, illumination laser on. The field of view is 500 μm × 416 μm.
